# The increasing incidence and prevalence of hypereosinophilic syndrome in the United Kingdom

**DOI:** 10.1002/iid3.495

**Published:** 2021-07-22

**Authors:** Gema Requena, John Logie, Daniel C. Gibbons, Jonathan Steinfeld, Melissa K. Van Dyke

**Affiliations:** ^1^ Epidemiology, Value Evidence and Outcomes, Global R&D, GSK London UK; ^2^ Real World Analytics, Value Evidence and Outcomes, Global Medical, GSK London UK; ^3^ Respiratory Research & Development, GSK Collegeville Pennsylvania USA; ^4^ Epidemiology, Value Evidence and Outcomes, Global R&D, GSK Collegeville Pennsylvania USA

**Keywords:** hypereosinophilic syndrome, incidence, prevalence, United Kingdom

## Abstract

**Introduction:**

Data on the burden of hypereosinophilic syndrome (HES) are limited. This study investigated the incidence and prevalence of HES using real‐world data from patients in the United Kingdom.

**Methods:**

Primary care data from the Clinical Practice Research Datalink were analyzed. The patients of interest were identified using medical codes specific for HES. Annual incidence rates and prevalence were estimated for the years 2010–2018 (inclusive) using patients observed for a minimum period of one year.

**Results:**

Between 2010 and 2018, 93 patients were identified with HES. During the study period the incidence of HES ranged from less than 0.04, 95% confidence interval (CI) (0.01–0.07) to 0.17, 95% CI (0.10–0.26) per 100,000 person‐years and the prevalence ranged from 0.15, 95% CI (0.10–0.25) to 0.89, 95% CI (0.74–1.09) cases per 100,000 persons. Sensitivity analyses varying the minimum observation period required to identify HES patients gave similar results.

**Conclusion:**

These results provide estimates of the burden of HES in the United Kingdom and indicate that whilst HES is a very rare disease, there is evidence that is increasingly being recorded in UK primary care.

## Graphical abstract

This study reports the annual incidence and prevalence of hypereosinophilic syndrome (HES) over an extended (9 year) period. Although HES remains a very rare condition, it is increasingly being recorded in UK primary care.

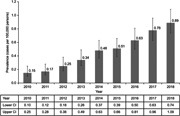

## 1 INTRODUCTION

Hypereosinophilic syndrome (HES) is a rare hematologic disorder without a known cause, characterized by the overproduction of eosinophils in the bone marrow,^1^, ^2^ and resulting in high blood eosinophil levels (eosinophilia).^2^ In patients with chronic or persistent eosinophilic tissue infiltration, the release of effector molecules by the activated eosinophils may result in inflammatory tissue damage and organ dysfunction,^3^ with dermatological, pulmonary, gastrointestinal, and cardiovascular symptoms all reported frequently.^1^, ^2^, ^3^, ^4^ Only after ruling out all other conditions associated with hypereosinophilia, such as infectious diseases, hematologic and neoplastic disorders, allergic disorders, organ‐specific disorders, or endocrine and immunologic disorders, can a diagnosis of HES be made.

Given that the diagnosis of HES is mainly a diagnosis of exclusion, we assumed that HES patients are primarily diagnosed by specialists, however, identification of HES patients within secondary databases is uncommon and we used a primary care database. The rationale for this was, first, because there was no specific code for HES in the International Classification of Diseases, version 10 at the time of the study, and it was encoded under the general term of “eosinophilia” (D72.1) which includes other types of eosinophilia (allergic, familial, secondary) or other related terms such as leucocytosis. Second, the identification of HES patients in an electronic healthcare record (EHR) database would require a complex unvalidated algorithm of laboratory results, inclusion and exclusion of many medical diagnosis codes, and potential treatments. And third, because we found specific Read codes for HES used in the Clinical Practice Research Datalink (CPRD) that could be used to identify these patients. While CPRD is a primary care database in the United Kingdom, we assumed that primary care physicians would only record a HES code in the patient file following a specialist's diagnosis. As a result of the rarity of HES, reliable estimates of the incidence and/or prevalence of HES are not readily available, and often meaningful estimates cannot be derived from publications based on case reports or small series of patients.

This study reports estimates of incidence and prevalence of HES in the United Kingdom using recent data from the CPRD, the world's largest database of anonymized, longitudinal primary care medical records.

## 2 METHODS

Data were drawn from two CPRD databases (Aurum and GOLD) containing primary care data captured from representative samples of general practices from across the United Kingdom but using different electronic medical record systems.^4^

There are different subtypes, and according to the 2016 World Health Organization classification of eosinophilic disorders, chronic eosinophilic leukemia (CEL) not otherwise specified, may be considered a subtype of HES as well.^5^ Thus the study population was identified as patients observed for a minimum of 1 year, with medical codes specific for HES and CEL available in these databases (GOLD Read codes: D403500 [Hypereosinophilic syndrome] and B651000 [Chronic eosinophilic leukaemia]; Aurum MEDCodeIDs: 207428016 [Hypereosinophilic syndrome], 932121000006116 [Hypereosinophilic syndrome] and 289895014 [Chronic eosinophilic leukaemia]).

Annual incidence rates and prevalence were estimated for the years 2010–2018, inclusive, based on an assumption that patients with existing, diagnosed, HES would be identified if they were followed for at least 365 days within CPRD. We consider it likely that a clinically significant condition such as HES would be recorded at least once in a patient's primary care records within the first 12 months following diagnosis (or within the first 12 months of registration for patients with existing HES who joined a new general practice). In prevalence analyses, this assumes that all existing, diagnosed, HES cases can be identified in a population followed for at least 365 days. For calculations of incidence, the assumption means that patients with no records of HES within the first 365 days of observation were considered disease‐free and hence “at risk” of developing HES in the future.

Incidence rates were calculated as the number of new cases of HES within a calendar year during the total “time at risk” of the eligible population within that calendar year. Under the assumption that patients cannot be reliably separated into incidence and prevalent cases during the first 365 days of observation, time at risk is considered to start 365 days after first registration in CPRD, and to end on the earliest date from the date of first HES diagnosis, the last day of observation, or the 31st December of the current year. Incidence rates were expressed per 100,000 person‐years, with 95% confidence intervals (CIs) calculated by the exact method as described by Ulm^6^ and Dobson et al.^7^ For calculations of prevalence, HES patients were identified from the population of patients who were continually registered for the full calendar year of interest; their HES diagnosis could occur either during, or at any time before, the calendar year of interest. Prevalence was expressed as the number of cases per 100,000 registered patients, with 95% CI calculated by the exact (Clopper–Pearson) method as described by Fleiss et al.^8^

The impact of the assumption that 365 days is sufficient to identify all existing, diagnosed patients, was tested in additional sensitivity analyses for both incidence and prevalence, by applying a requirement of a minimum observation period of 730 days (2 years) or 1035 days (3 years) to identify existing prevalent cases. Besides, a sensitivity analysis with a shorter observation period of 6 months was conducted to assess if changing the observation period, it resulted in the identification of additional patients.

To assess the possibility that patients' diagnoses may not have been recorded using the specific Read codes for HES in the early years of the study if these codes were not widely known or adopted, we summarized the medical records of patients in the 365 days before their first record of HES to determine if there were changes in the use of general codes over time.

The study protocol was approved by the CPRD Independent Scientific Advisory Committee, number 18_242AMnA. CPRD collects anonymized patient data from a network of GP practices across the United Kingdom. They process the data in accordance with the ICO's anonymization code of practice. CPRD does not receive or hold patient identifiers including name, full date of birth, postcode, and NHS number. Identifiers are removed before transfer of data to CPRD to protect patient confidentiality. CPRD obtains annual research ethics approval from the UK's Health Research Authority Research Ethics Committee (East Midlands—Derby, REC reference number 05/MRE04/87) to receive and supply patient data for public health research.

## 3 RESULTS

**Figure 1:**
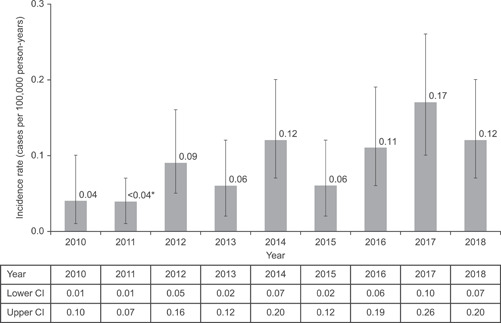
Annual incidence of hypereosinophilic syndrome in the UK Clinical Practice Research Datalink from 2010 to 2018. CI, confidence interval. *In accordance with the standard Clinical Practice Research Datalink policy, no cell counts with less than 5 (or rates based on them) should be shared

**Figure 2:**
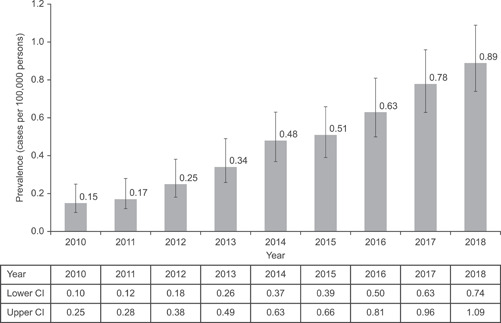
Annual prevalence of hypereosinophilic syndrome in the UK Clinical Practice Research Datalink from 2010 to 2018. CI, confidence interval

During the study period, a total of 93 patients were identified with a first diagnosis for HES from a total population of 22 million patients. Among these, 57% of patients were male, with a mean (*SD*) age of 57.0 (19.3) years and a range of 13–95 years. Of these, 12 patients had a diagnosis of CEL; they were slightly older (average age of 67 years) and predominantly male (92%). The overall annual incidence rate of patients diagnosed with HES ranged from less than 0.04, 95% CI (0.01–0.07) to 0.17, 95% CI (0.10–0.26) per 100,000 person‐years between 2010 and 2018 (Figure 1). The overall annual prevalence ranged from 0.15, 95% CI (0.10–0.25) to 0.89, 95% CI (0.74–1.09) cases per 100,000 persons during this same period (Figure 2). The sensitivity analyses with observation periods of 6 months, 2 and 3 years yielded similar results. The most common diagnosis class in the 365 days before first HES/CEL diagnosis across all years was “Disease of white blood cells,” which was recorded for a similar proportion of patients in 2010–2014 (42.5% of patients [*n* = 40]) and 2015–2018 (39.6% [*n* = 53]).

## 4 DISCUSSION

This study is the first to report the annual incidence and prevalence of HES over an extended (9 year) period and both increased over time. The only published data about the incidence of HES in the general population that we are aware of is a previous study from the United States that evaluated the incidence of myeloproliferative HES/chronic eosinophilic leukemia between 2001 and 2005. From these data, the authors extrapolated the incidence of all types of HES providing broad estimates to be in the range of 0.018–0.036 to 0.18–0.36 per 100,000 person‐years.^9^

Our findings indicate that although HES remains a very rare condition, it is increasingly being recorded in UK primary care. We investigated several possible explanations for these findings.

It remains possible that, since patients are likely to be diagnosed by specialist physicians, there might be an underreporting of HES in a primary care database. However, in the United Kingdom, GPs are the gatekeeper to secondary and tertiary healthcare and it remains unlikely that significant diagnoses affecting the ongoing management of patients, such as HES, would not be communicated back to primary care, and therefore recorded in the patient's general practitioner record. Given that HES is a rare and complex diagnosis that can only be made by specialists after ruling out other secondary causes of hypereosinophilia, GPs are unlikely to use specific “HES” codes without being informed by specialist care. We, therefore, believe that the use of these specific codes in primary care records is likely to be reliable. Moreover, the availability of these codes allows a specific case definition to be applied within CPRD.

Results remained unchanged in sensitivity analyses where we increased the minimum observation time required to identify existing, diagnosed, HES cases from 1 to 2 and 3 years or reduced this time to 6 months. Again, this suggests that the rarity of HES, and the temporal trends seen, are not due to the analyses failing to identify infrequent transfers of details from elsewhere in the health system into a patient's primary care records, or the misclassification of existing prevalent cases as incident patients in later years. The finding that the duration of prior time within the database does not affect prevalence or incidence rates would suggest that the results are not a result of an artifact such as the availability of longer durations of medical history in later years. The data, however, do not allow us to separate the possibilities that the increase in the recorded diagnosis of HES observed over time resulted from improved recognition and coding of HES in clinical practice from a true underlying increase in the incidence and/or prevalence of this condition.

New definitions and classifications of HES have been developed, potentially increasing the awareness of this disease and its diagnosis and management among specialists.^10^ In addition, the recent development of treatments for conditions with similar presentation may have led to increase the referrals to specialists. It remains possible that, in the early years of this study, patients' diagnosis may not have been recorded using the specific Read codes for HES if these codes were not widely known or adopted, even if a diagnosis was shared from secondary care. However, we did not find evidence that a general code preceded a HES diagnosis more frequently in the early years of this study (e.g., “Diseases of white blood cells” recorded for 42.5% of patients (*n* = 40) in the period from 2010 to 2014 vs. 39.6% for 2015–2018 (*n* = 53), although based on small numbers of patients.

Finally, despite using specific codes to identify patients diagnosed with HES, we cannot be sure that these codes define properly all HES subtypes, thus the CEL code used might refer to other types of CEL and not just the HES subtype. The number of patients identified with CEL was very small, with minimal impact in the study results. Nevertheless, the use of clinical codes for identifying patients in EHR database studies posed a challenge in the inclusion/omission of the codes employed and it may be a study limitation.

This study highlights the utility of using routine electronic medical records systems to describe the epidemiology of rare diseases such as HES. These data do not intend to provide a pathology review of HES or its subtypes but provide the unique value of enumerating the population from which patients are drawn and allow the absolute burden of HES to be described. Results show a clear increase in the recording of HES within the UK, although this remains a very rare condition. The exact contributions of true increases in underlying diagnosis from changes in recognition and/or recording remain unclear. Nevertheless, we believe this information contributes to an important gap in information on the incidence and prevalence of this rare disease in the literature.

## CONFLICT OF INTERESTS

Gema Requena, John Logie, Daniel C. Gibbons, Jonathan Steinfeld, and Melissa K. Van Dyke are employees of GSK and own stocks/shares in GSK. Editorial support was provided by Kerry Knight, PhD, at Fishawack Indicia Ltd., UK, and was funded by GSK.

## AUTHOR CONTRIBUTIONS

Gema Requena, John Logie, Melissa K. Van Dyke contributed to the conception and design of the analysis, acquisition of the data, and analysis and interpretation of the data. Jonathan Steinfeld contributed to the conception and design of the analysis and analysis and interpretation of the data. Daniel C. Gibbons contributed to the analysis and interpretation of the data. All authors critically revised the manuscript for intellectual content, gave final approval of the version to be published, and agreed to be accountable for all aspects of the work.

## Data Availability

GSK makes available anonymized individual participant data and associated documents from interventional clinical studies which evaluate medicines, upon approval of proposals submitted to https://www.clinicalstudydatarequest.com. To access data for other types of GSK sponsored research, for study documents without patient‐level data and for clinical studies not listed, please submit an enquiry via the website. The data that support the findings of this study are available from UK CPRD. Researchers can apply to access CPRD data; access is conditional on approval by the Independent Scientific Advisory Committee.

## References

[1] Ackerman SJ , Bochner BS . Mechanisms of eosinophilia in the pathogenesis of hypereosinophilic disorders. Immunol Allergy Clin North Am. 2007;27(3):357‐375. 17868854 10.1016/j.iac.2007.07.004 PMC2064859

[2] Curtis C , Ogbogu P . Hypereosinophilic syndrome. Clin Rev Allergy Immunol. 2016;50(2):240‐251. 26475367 10.1007/s12016-015-8506-7

[3] Valent P , Klion AD , Horny HP , et al. Contemporary consensus proposal on criteria and classification of eosinophilic disorders and related syndromes. J Allergy Clin Immunol. 2012;130(3):607‐612e609. 22460074 10.1016/j.jaci.2012.02.019 PMC4091810

[4] Wolf A , Dedman D , Campbell J , et al. Data resource profile: Clinical Practice Research Datalink (CPRD) Aurum. Int J Epidemiol. 2019;48:1740. 10.1093/ije/dyz034 30859197 PMC6929522

[5] Gotlib J . World Health Organization‐defined eosinophilic disorders: 2017 update on diagnosis, risk stratification, and management. Am J Hematol. 2017;92(11):1243‐1259. 29044676 10.1002/ajh.24880

[6] Ulm K . Simple method to calculate the confidence interval of a standardized mortality ratio (SMR). Am J Epidemiol. 1990;131(2):373‐375. 2296988 10.1093/oxfordjournals.aje.a115507

[7] Dobson AJ , Kuulasmaa K , Eberle E , Scherer J . Confidence intervals for weighted sums of Poisson parameters. Stat Med. 1991;10(3):457‐462. 2028128 10.1002/sim.4780100317

[8] Fleiss JL , Levin B , Paik MC . Statistical Inference for a Single Proportion. Statistical Methods for Rates and Proportions. 3rd ed. New York: John Wiley & Sons; 2003.

[9] Crane MM , Chang CM , Kobayashi MG , Weller PF . Incidence of myeloproliferative hypereosinophilic syndrome in the United States and an estimate of all hypereosinophilic syndrome incidence. J Allergy Clin Immunol. 2010;126(1):179‐181. 20639012 10.1016/j.jaci.2010.03.035 PMC5781228

[10] Kahn JE , Groh M , Lefevre G . (A Critical Appraisal of) Classification of hypereosinophilic disorders. Front Med (Lausanne). 2017;4:216. 29259972 10.3389/fmed.2017.00216 PMC5723313

